# TNF-α and TNFR1 responses to recovery therapies following acute resistance exercise

**DOI:** 10.3389/fphys.2015.00048

**Published:** 2015-02-18

**Authors:** Jeremy R. Townsend, Jay R. Hoffman, Maren S. Fragala, Adam R. Jajtner, Adam M. Gonzalez, Adam J. Wells, Gerald T. Mangine, David H. Fukuda, Jeffrey R. Stout

**Affiliations:** Department of Education and Human Performance, Institute of Exercise Physiology and Wellness, University of Central FloridaOrlando, FL, USA

**Keywords:** muscle damage, immune function, cytokines, athletic training, TNF-α

## Abstract

The purpose of this investigation was to compare the effect of two commonly used therapeutic modalities (a) neuromuscular electrical stimulation (NMES) and (b) cold water immersion (CWI) on circulating tumor necrosis factor alpha (TNF-α) and monocyte TNF-α receptor (TNFR1) expression following intense acute resistance exercise and subsequent recovery. Thirty (*n* = 30) resistance trained men (22.5 ± 2.7 y) performed an acute heavy resistance exercise protocol on three consecutive days followed by one of three recovery methods (CON, NMES, and CWI). Circulating TNF-α levels were assayed and TNFR1 expression on CD14+ monocytes was measured by flow cytometry measured PRE, immediately post (IP), 30-min post (30M), 24 h post (24H), and 48 h post (48H) exercise. Circulating TNF-α was elevated at IP (*p* = 0.001) and 30M (*p* = 0.005) and decreased at 24H and 48H recovery from IP in CON (*p* = 0.015) and CWI (*p* = 0.011). TNF-α did not significantly decrease from IP during recovery in NMES. TNFR1 expression was elevated (*p* < 0.001) at 30M compared to PRE and all other time points. No significant differences between groups were observed in TNFR1 expression. During recovery (24H, 48H) from muscle damaging exercise, NMES treatment appears to prevent the decline in circulating TNF-α observed during recovery in those receiving no treatment or CWI.

## Introduction

Tumor necrosis factor-α (TNF-α) is a multifunctional cytokine involved in the regulation of inflammation and tissue injury. Among its various functions, it is most noted for its pro-inflammatory role in tissue degradation (Li and Reid, 2001; Peterson et al., 2006; Zaldivar et al., 2006). As an early mediator in muscle damage, TNF-α can be synthesized by several immune and nervous cells and is rapidly released in the blood by circulating monocytes and in skeletal muscle by invading macrophages after exercise-induced muscle damage (Hirose et al., 2004; Zaldivar et al., 2006). Furthermore, TNF-α can induce both necrosis and apoptosis of myocytes through intracellular signaling pathways (Hardin et al., 2008) which accompanies a decline in muscle contractile properties and diminished performance (Cassatella, 1995; Hardin et al., 2008).

Strenuous exercise often results in considerable muscle damage that may impact subsequent athletic performance (Crameri et al., 2007). Thus, various recovery modalities are often used by athletes as a means to counteract the inflammatory response which accompanies the potentially damaging effects of intense exercise. Cold water immersion (CWI) is one of the most common recovery modalities used by athletes to expedite muscle repair and recovery (Barnett, 2006; Rice et al., 2008; Bleakley et al., 2012).

While previous literature has shown beneficial results of CWI such as decreasing indirect markers of muscle damage as well as attenuating pro-inflammatory cytokine release, some studies show little to no effect on blood markers and immune responses (Bleakley et al., 2012; Leeder et al., 2012; Tseng et al., 2013). While there is evidence that CWI is effective in decreasing leukocytosis (Tseng et al., 2013), to date, research on the effects of CWI on inflammatory cells following intense exercise is limited (Leeder et al., 2012; Tseng et al., 2013).

Neuromuscular electrical stimulation (NMES) is another therapeutic modality used to facilitate rehabilitation and recovery after muscular trauma (Peake et al., 2008; Babault et al., 2011). Current literature examining the benefit NMES is equivocal, revealing both positive and neutral effects on measures of muscle strength and power, as well as pain modulation (Miller et al., 2000; Peake et al., 2008; Babault et al., 2011). One possible mechanism for the benefit of NMES is an increase in blood flow (Babault et al., 2011), which could possibly lead to an increase in recruitment of leukocytes to damaged tissue, potentially accelerating the muscle regeneration process. While currently unknown, it is possible that NMES may also elicit unique effects on specific immune cells and cytokines.

The purpose of the present investigation was to investigate the efficacy of CWI and NMES on the cellular interaction between circulating TNF-α levels and its receptor expression (TNFR1) on human monocytes in response to heavy resistance exercise. Since circulating concentrations of cytokines exert their effects through binding to a receptor, TNFR1 was included in our measurement. The assessment of how recovery modalities mediate the inflammatory response may provide additional insight to muscle recovery processes and subsequent performance. We hypothesized that CWI would mitigate circulating TNF-α and TNFR1 expression while NMES would not attenuate TNF-α and TNFR1 on circulating monocytes following the heavy resistance training protocol and subsequent bouts of resistance exercise.

## Methods

### Participants and design

Thirty resistance-trained men (22.5 ± 2.7 y, 1.74 ± 0.12 m, 83.4 ± 6.9 kg) participated in this study and were randomly divided into one of three groups: Control (CON), neuromuscular electrical stimulation (NMES), and cold water immersion (CWI), by use of a random number generator. Participants were required to have a minimum of 1 year of resistance training experience to participate, particularly in the squat exercise, and were not permitted to use additional nutritional supplements or recovery strategies while enrolled in the study. The University Institutional Review Board approved the research protocol which was in accordance with the Declaration of Helsinki and each participant gave his informed consent.

### Methodology

**Figure 1:**
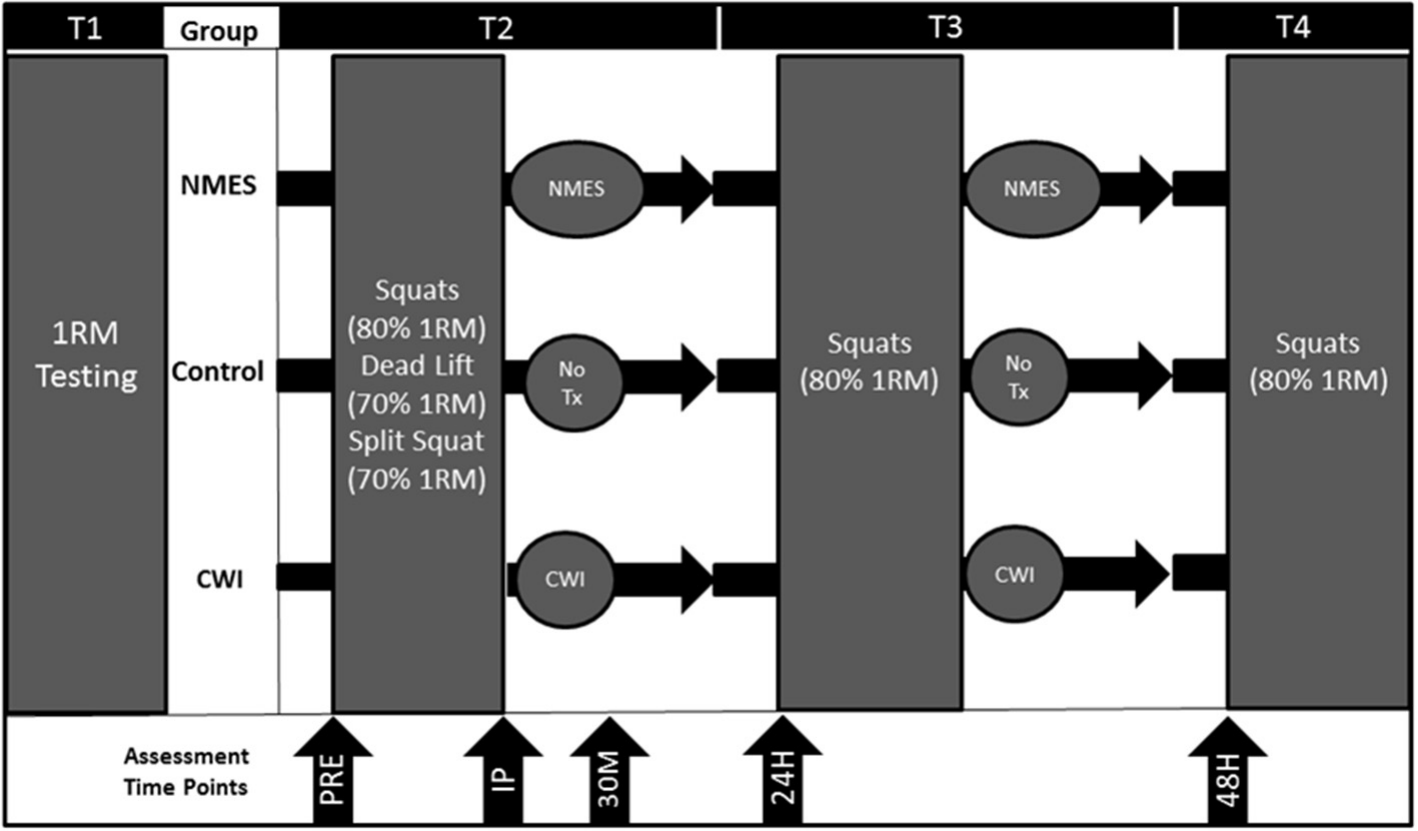
**Study design for control (CON), neuromuscular electrical stimulation (NMES), and cold water immersion (CWI)**. PRE = Pre-exercise. IP = Immediately post-exercise. 30M = 30 min post-exercise. 24H = 24 h post-exercise. 48H = 48 h post-exercise. 1RM = 1-repetiton maximum. Tx = Treatment.

Participants reported to the lab on four separate occasions. On the first visit (T1), participants were tested for maximal strength [one repetition-maximum (1-RM)] on the barbell back squat, dead lift, and barbell split squat exercises. Prior to the second visit (T2), participants were instructed to refrain from exercising for 72 h. The 1RM session was completed no more than a week before the T2 visit. At T2, participants performed four sets of no more than 10 repetitions for each set until failure of the squat (80% 1-RM), dead lift (70% 1-RM), and barbell split squat (70% 1-RM) exercises with 90 s rest intervals between each set and exercise. At T3 and T4, squats (4 sets of 10 repetitions at 80% 1RM) were performed (Figure 1).

### Neuromuscular electric stimulation therapy (NMES)

Participants randomly assigned to NMES were provided with 24 min of electrical stimulation immediately following the post-exercise blood draw (T2) or post-exercise (T3) using a commercially available product (Compex Performance US, DJO, Vista, CA). Participants were asked to lay supine with three electrodes placed on the quadriceps muscles of each leg. Specifically, one large electrode with a negative charge was placed at the most proximal point of the upper leg, while two small electrodes with positive charge were placed on the belly of the vastus lateralis and vastus medialis. The unit was set to the manufacturer's established recovery mode. The investigator adjusted the stimulation intensity to a level as high as possible before the participant felt unwarranted discomfort. The intensity administered to each participant was noted by the research investigator, and was used for the subsequent recovery intervention period (T3), similar to previously published methods (Vanderthommen et al., 2007). The treatment protocol consisted of nine sequences, with the first three stages lasting for 2 min, and the remaining six for 3 min. Frequency of contraction started at 9 Hz, stepping down 1 Hz per stage to 1 Hz.

### Cold water immersion

Immediately post-exercise participants in the CWI group were provided a 22.9 cm high ice bath with a temperature that was maintained at 10–12°C immediately following a post-exercise blood draw (T2) or immediately post-exercise (T3). Participants were required to fully submerge their lower body into the water up to their umbilicus for 10-min.

### Blood measurements

During the T2 experimental session, blood samples were obtained pre-exercise for baseline measurement (PRE), immediately post-exercise (IP), and 30 min post-exercise (30M). During the T3 and T4 experimental sessions, blood samples were obtained pre-exercise marking 24 h post-exercise (24H), and 48 h post-exercise (48H). During T2, all blood samples were obtained using a 20-gauge Teflon cannula placed in a superficial forearm vein kept patent with isotonic saline. PRE blood samples were drawn following a 15-min equilibration period prior to exercise. IP blood samples were taken within 1 min of exercise cessation. Each participant's blood samples were obtained at the same time of day during each session.

Blood samples were collected into two Vacutainer® tubes, one containing no anti-clotting agent and the second containing K_2_ EDTA. The blood in the first tube was allowed to clot at room temperature for 30 min and subsequently centrifuged at 3000 × g for 15 min along with the remaining whole blood from the second tube. The resulting plasma and serum was placed into separate 1.8-mL microcentrifuge tubes and frozen at −80°C for later analysis.

### Biochemical analysis

Creatine Kinase (CK) was analyzed with the use of a spectrophotometer and a commercially available enzymatic kit (Sekisui Diagnostics, Charlottetown, PE, Canada) per manufacturer's instructions. Myoglobin concentrations were determined using enzyme-linked immunosorbent assays (ELISA) (Calbiotech, Spring Valley, CA). Determination of serum immunoreactivity values was determined using a BioTek Eon spectrophotometer (BioTek, Winooski, VT, USA). All samples were run in duplicate with a mean intra-assay variance of 2.6% for CK and 5.73% for myoglobin. Coefficient of variation for each hemoglobin assay was 3.73% for and 0.65% for hematocrit.

Serum samples were assayed for concentrations of TNF-α, using a cytokine assay (Millipore Milliplex, cat no. HCYTOMAG-60K; Billerica, MA) on a MAGPIX instrument (Luminex Technologies; Luminex, Austin, TX) according to the manufacturer's instructions. All samples were run in duplicate with a mean intra-assay variance of 8.36% for TNF-α.

### Cell staining

Erythrocytes were lysed from 350 μl of whole blood (Pharm Lyse; BD Biosciences, Franklin Lakes, NJ) within 30 min of collection. Samples were then washed in staining buffer containing 1 × phosphate-buffered saline containing fetal bovine serum (FBS) by centrifugation and aspiration. This process was repeated three times. Leukocytes were then resuspended in 100 μl Pharminigen stain buffer (BD Biosciences, Franklin Lakes, NJ). Direct staining methods were used to label CD14 and CD120a (TNFR1). Fluorescein isothiocyanate (FITC) conjugated to anti-CD120a (H398, IgG2 α; AbDSerotec, Raleigh, NC) and PerCP Cy5.5 conjugated anti-CD14 (M5E2; BD Pharminigen) were used in the receptor labeling process. Surface staining was performed by adding 10 μl of directly conjugated FITC-anti-CD120a, 5 μl of directly conjugated PerCP Cy5.5-anti-CD14 to the cell suspension and incubating in the dark for 30 min at 20°C. Cells were resuspended in 1.0 ml of stain buffer for flow cytometry analysis.

### Flow cytometry

Flow cytometry analysis of stained cells was performed on a C6 Accuri Flow Cytometer (BD Biosciences); equipped with BD Accuri analysis software (BD Biosciences). Forward and side scatter along with four fluorescent channels of data were collected using two lasers providing excitation at 488 and 640 nm. Monocytes were determined by initial gating based on forward and side scatter, followed by gating for CD14+ cells as also described by Tallone et al. (2011). A minimum of 10,000 events, defined as CD14+ monocytes, were obtained with each sample. Analysis of monocyte subpopulations was completed by quadrant analyses, in which CD14 was compared with CD120a. Mean fluorescence of CD120a was recorded, representing the mean density of receptors per cell.

### Statistical analysis

A repeated measures analysis of variance (ANOVA) was used to analyze both TNF-α and TNFR1 data. If an interaction was found, follow-up analyses included One-Way repeated measures ANOVAs and Tukey *post-hoc* comparisons. Prior to analysis all data was assessed to ensure normal distribution, homogeneity of variance and sphericity. Dietary compositions between groups were analyzed using an unpaired *t*-test. Results were considered significant at an alpha level of *p* = 0.05. All data are reported as mean ± SD. Data were analyzed using SPSS v20 software (SPSS Inc., Chicago, IL, USA).

## Results

Participants assigned to the CWI, NMES, and CON groups did not differ in in any physical characteristics. Participants were experienced, resistance trained individuals having an average resistance training history of 7.9 ± 3.3 years and an average squat 1RM of 150.2 ± 22.4 kg. Additionally, the exercise load presented a similar stress to all groups in terms of total volume and number of repetitions performed. Muscle damage corresponded with significant elevations in CK from PRE (103.4–166.3 U/L) to 24H (290%, 468.9–677.1 U/L) and 48H (271% 424.6–612.5 U/L) post-T2 and myoglobin from PRE (23.3–35.5 ng/mL) to IP (192%, 63.8–114.8 ng/mL) and 30P (357%, 89.3–180.6 ng/mL), as reported previously (Jajtner et al., 2014).

CK and myoglobin responses to the protocol did not appear to be affected by NMES or CWI treatments. Analysis of the participants' dietary recall revealed no differences in total caloric or protein intake (g and g•kg^−1^ body mass) between groups on days of T2 and T3.

### Circulating TNF-α levels

**Figure 2:**
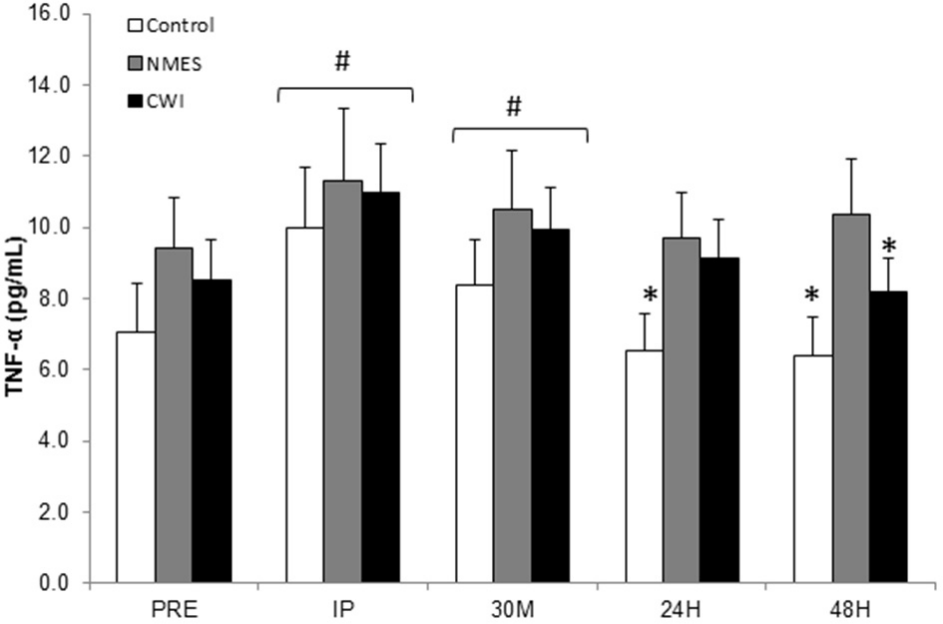
**Circulating TNF-α response to acute heavy resistance exercise and subsequent recovery in control (CON), neuromuscular electro stimulation (NMES) and cold water immersion (CWI), groups at Pre-exercise (PRE), Immediately post-exercise (IP), 30-min post-exercise (30M), 24 h post-exercise (24H), and 48 h post-exercise (48H) ^#^ Significantly elevated from PRE (*p* < 0.05)**. ^*^ Significantly (*p* ≤ 0.05) lower than IP.

No between-group differences were noted in TNF-α at PRE (8.3 ± 4.2 pg/mL). The Two-Way time × group repeated measures ANOVA for TNF-α indicated a significant time effect (*F* = 8.68, *p* < 0.001), but no significant main effect for group (*p* > 0.05), or time × group interaction (*p* > 0.05). With all groups combined, circulating TNF-α was elevated at IP (10.8 ± 5.3 pg/mL; *p* = 0.001) and at 30M (9.6 ± 4.3 pg/mL; *p* = 0.005) compared to PRE concentrations (Figure 2). TNF-α concentrations were in similar physiological range to other resistance trained males (Kraemer et al., 2014).

Since treatments were not administered until after IP, a separate repeated measures was run from IP to determine the effect of each treatment. When compared to IP values, TNF-α was significantly lower at 24H (*p* = 0.015) and 48H (*p* = 0.001) in CON. CWI was significantly lower (*p* = 0.011) at 48H compared to IP values.

### TNFR1

**Figure 3:**
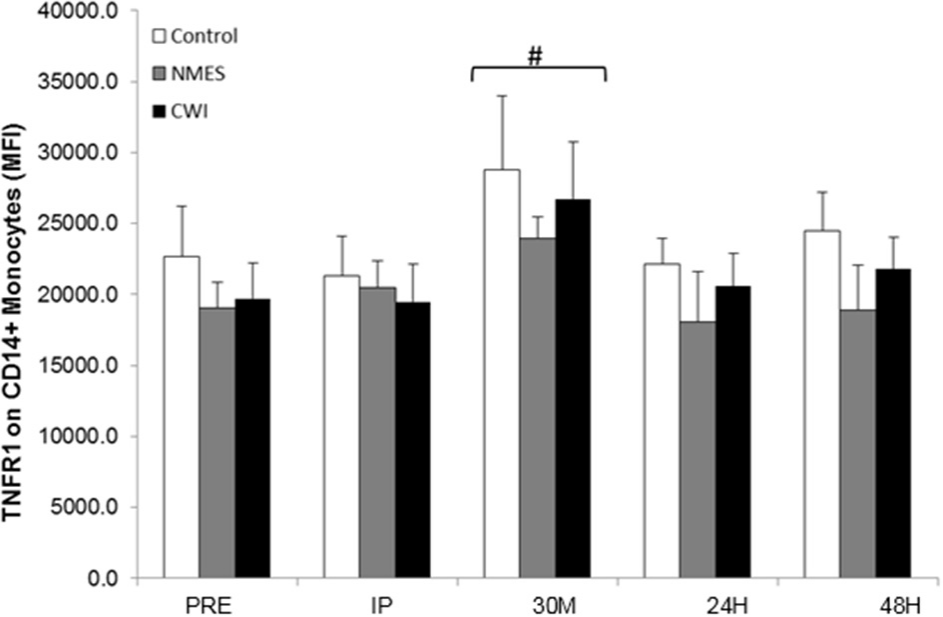
**TNFR1 [expressed as Mean Fluorescence Intensity (MFI)] on CD14+ monocytes response to acute heavy resistance exercise and subsequent recovery in control (CON), neuromuscular electro stimulation (NMES) and cold water immersion (CWI), groups at Pre-exercise (PRE), Immediately post-exercise (IP), 30-min post-exercise (30M), 24 h post-exercise (24H), and 48 h post-exercise (48H) ^#^ Significantly elevated from PRE (*p* < 0.05)**.

One hundred percent of CD+14 monocytes expressed TNFR1. The Two-Way × group repeated measures ANOVA for TNFR1 density (expressed as relative fluorescence) indicated a significant time effect (*F* = 4.25, *p* = 0.023), but no main effect for group (*p* > 0.05), or time × group interactions (*p* > 0.05). TNFR1 levels were significantly elevated (*p* < 0.01) at 30M compared to all other time points (Figure 3).

## Discussion

The main findings of this investigation were that TNF-α was elevated immediately following acute heavy resistance exercise, and decreased at 24 h and 48 h into recovery in those receiving no treatments. Treatment with NMES appeared to prevent the recovery decline in circulating TNF-α compared to the CON group. In addition, TNFR1 expression on CD14+ monocytes appears to be elevated 30-min following acute heavy resistance exercise, and did not appear to be affected by treatment during the recovery period.

The increase in circulating TNF-α following heavy resistance exercise is consistent with findings reported by others. Brenner et al., found that resistance exercise increased TNF-α to a greater extent than moderate aerobic or maximal cycling (Brenner et al., 1999). Conversely, some investigations have found no change in TNF-α, Smith et al. (2000), Peake et al. (2006), and in one study a decrease in TNF-α was observed (Hirose et al., 2004). However, studies which saw no elevation in TNF-α recruited individuals without prior resistance training experience and employed only eccentric movements (Smith et al., 2000; Buford et al., 2009). Another study, which observed a decrease in circulating TNF-α, used only eccentric elbow flexion (Hirose et al., 2004). Therefore, it is likely that resistance-trained individuals performing heavy dynamic resistance exercise experience a more pronounced TNF-α response following an acute exercise bout than non-trained individuals. The elevated circulating TNF-α levels in response to heavy resistance exercise is an important pro-inflammatory event in the tissue repair process (Freidenreich and Volek, 2012). Resistance-trained individuals possibly elicit an increased immunological response to exercise and when coupled with large amount of muscle mass involved in this type of exercise, acutely produce more pro-inflammatory cytokines (Leeder et al., 2012).

Without treatment, circulating TNF-α returned to baseline at 24H and 48H into recovery. Treatment with NMES resulted in circulating TNF-α concentrations that did not return to baseline values at 24H and 48H into recovery while. To the authors' knowledge, this is the first study to examine the effects of NMES on the immune response to heavy resistance training. While used commonly in therapy settings for pain modulation, there is little evidence in the literature that NMES is an effective means of recovery following strenuous exercise (Vanderthommen et al., 2007; Peake et al., 2008).

NMES is believed to function by initiating muscular contractions that accelerate the blood flow to the stimulation site, which increases the rate of lactate and other metabolic byproduct removal (Babault et al., 2011). Therefore, in the present study, NMES treatment may have provided a constant stimulus for tissue breakdown and removal. The nature of NMES stimulating muscle contractions may have evoked a continuous elevation of pro-inflammatory cytokines during the recovery period as TNF-α has been shown to be expressed from injured skeletal muscle (Tseng et al., 2013). The CWI group was elevated at IP and returned to baseline at 48 h post-exercise. Since there is evidence that CWI is effective in decreasing leukocytosis (Tseng et al., 2013), it is possible that cryotherapy may prevent or even delay the repair process.

Our data demonstrate an exercise-induced elevation in TNFR1 on CD14+ monocytes at 30M following heavy resistance exercise which is supports previously published data (Townsend et al., 2013). However, TNFR1 expression on CD14+ monocytes was not significantly different between any of the interventions. While not significant, TNFR1 expression on CD14+ monocytes in NMES was lower on average than expression in CON and CWI during recovery. This lower expression corresponded to time points where circulating TNF-α was elevated in the NMES group in our study. Previous studies have also reported that receptor expression and serum concentration cytokine patterns may not coincide (Peake et al., 2006; Zaldivar et al., 2006). Potentially, elevations in circulating cytokines may be saturating their receptors and thus reducing subsequent expression (Fragala et al., 2011). Furthermore, secretion of TNF-α cannot solely be attributed to immune cells (Li and Reid, 2001), so it is possible that NMES modulates the presence of TNFR1 on monocytes differently than serum concentration TNF-α and the observed increase in circulating TNF-α may have been attributed to muscle cytokine release. Nevertheless, there is currently little research on TNFR1 expression on leukocytes in response to strenuous resistance exercise protocols, and further investigations are needed aimed at determining how exercise modulates specific immune cells and the implications of their response.

Although inflammation has been commonly associated with detrimental consequences, pro-inflammatory processes are essential for subsequent muscle repair and regeneration (Li and Reid, 2001; Peterson et al., 2006; Crameri et al., 2007; Urso, 2013). In the tissue repair cascade, early responding inflammatory cells, mainly circulating monocytes (Peake et al., 2005) and tissue-embedded macrophages secrete several pro-inflammatory cytokines which stimulate satellite cell activation (Mackey et al., 2007). Thus, interventions that attenuate or block inflammation may actually hinder or delay the repair processes (Mackey et al., 2007; Urso, 2013). Nevertheless, prior studies have shown that acute anti-inflammatory treatments may allow for improved subsequent muscular contractile and structural function (Grounds and Torrisi, 2004; Piers et al., 2011). Thus, it appears that there may be specific time points following acute damage in which anti-inflammatory interventions are most beneficial to muscle contractile function and repair, however these time frames have yet to be identified (Urso, 2013).

In the present study, therapies were implemented immediately following exercise, which resulted in elevated circulating TNF-α concentration during recovery compared to controls. While this is the first study to report the effect of NMES on circulating TNF-α and TNFR1 expression following heavy resistance exercise, it is important to consider that the cytokine response to exercise stress and subsequent muscle disruption is complex and in constant flux (Cassatella, 1995; Peake et al., 2005; Bleakley et al., 2012). Whereas the present study examined TNF-α and its receptor as a possible mechanism to explain the efficacy of recovery modalities, it is important to note that several additional pro-and anti-inflammatory cytokines and immune markers (IL-1β, IL-6, IL-10) contribute to the tissue repair processes. However, the aim of the current investigation was to provide additional insight to an important subset of immune cells in response to acute resistance exercise. Further study of additional cells and cytokines are needed to fully elucidate the mechanisms behind therapeutic modalities in tissue recovery.

In conclusion, it appears that CWI and NMES therapies do not attenuate circulating TNF-α during recovery compared to control conditions, as had been hypothesized. However, it seems that NMES is potentially attenuates TNFR1 expression more than CWI. These data contribute new insights on how recovery modalities affect the immune response to muscle recovery from resistance exercise.

## Author contributions

Conception and design of research: JT, JH, MF, AJ, AG, JS. Acquisition of data: JT, AJ, AG, AW. Data analysis and interpretation: JT, JH, MF, AJ, AG, GM, AW, JS, DH. Drafting of manuscript and manuscript approval: JT, JH, MF, AJ, AG, GM, JS, DH.

### Conflict of interest statement

The authors declare that the research was conducted in the absence of any commercial or financial relationships that could be construed as a potential conflict of interest.

## References

[B1] Babault N. Cometti C. Maffiuletti N. A. Deley G. (2011). Does electrical stimulation enhance post-exercise performance recovery? Eur. J. Appl. Physiol. 111, 2501–2507. 10.1007/s00421-011-2117-7 21847574

[B2] Barnett A. (2006). Using recovery modalities between training sessions in elite athletes. Sports Med. 36, 781–796. 10.2165/00007256-200636090-00005 16937953

[B3] Bleakley C. Glasgow P. Webb M. (2012). Cooling an acute muscle injury: can basic scientific theory translate into the clinical setting? Br. J. Sports Med. 46, 296–298. 10.1136/bjsm.2011.086116 21677317

[B4] Brenner I. Natale V. Vasiliou P. Moldoveanu A. Shek P. Shephard R. (1999). Impact of three different types of exercise on components of the inflammatory response. Eur. J. Appl. Physiol. Occup. Physiol. 80, 452–460. 10.1007/s004210050617 10502079

[B5] Buford T. W. Cooke M. B. Willoughby D. S. (2009). Resistance exercise-induced changes of inflammatory gene expression within human skeletal muscle. Eur. J. Appl. Physiol. 107, 463–471. 10.1007/s00421-009-1145-z 19669788

[B6] Cassatella M. A. (1995). The production of cytokines by polymorphonuclear neutrophils. Immunol. Today. 16, 21–26. 10.1016/0167-5699(95)80066-2 7880385

[B7] Crameri R. Aagaard P. Qvortrup K. Langberg H. Olesen J. Kjær M. (2007). Myofibre damage in human skeletal muscle: effects of electrical stimulation versus voluntary contraction. J. Physiol. 583, 365–380. 10.1113/jphysiol.2007.128827 17584833 PMC2277245

[B8] Fragala M. S. Kraemer W. J. Mastro A. M. Denegar C. R. Volek J. S. Hakkinen K.. (2011). Leukocyte β2-adrenergic receptor expression in response to resistance exercise. Med. Sci. Sports Exerc. 43, 1422–1432. 10.1249/MSS.0b013e31820b88bc 21200338

[B9] Freidenreich D. J. Volek J. S. (2012). Immune responses to resistance exercise. Exerc. Immunol. Rev. 18, 8. 22876721

[B10] Grounds M. D. Torrisi J. (2004). Anti-TNFα (Remicade®) therapy protects dystrophic skeletal muscle from necrosis. FASEB J. 18, 676–682. 10.1096/fj.03-1024com 15054089

[B11] Hardin B. J. Campbell K. S. Smith J. D. Arbogast S. Smith J. Moylan J. S.. (2008). TNF-α acts via TNFR1 and muscle-derived oxidants to depress myofibrillar force in murine skeletal muscle. J. Appl. Physiol. 104, 694–699. 10.1152/japplphysiol.00898.2007 18187611

[B12] Hirose L. Nosaka K. Newton M. Laveder A. Kano M. Peake J.. (2004). Changes in inflammatory mediators following eccentric exercise of the elbow flexors. Exerc. Immunol. Rev. 10, 20. 15633588

[B13] Jajtner A. R. Hoffman J. R. Gonzalez A. M. Worts P. Fragala M. S. Stout J. R. (2014). Comparison of electrical stimulation versus cold water immersion treatment on muscle soreness following resistance exercise. J. Sport Rehabil. [Epub ahead of print]. 10.1123/jsr.2013-0113 24622577

[B14] Kraemer W. J. Hatfield D. L. Comstock B. A. Fragala M. S. Davitt P. M. Cortis C.. (2014). Influence of HMB supplementation and resistance training on cytokine responses to resistance exercise. J. Am. Coll. Nutr. 33, 247–255. 10.1080/07315724.2014.911669 25140763

[B15] Leeder J. Gissane C. van Someren K. Gregson W. Howatson G. (2012). Cold water immersion and recovery from strenuous exercise: a meta-analysis. Br. J. Sports Med. 46, 233–240. 10.1136/bjsports-2011-090061 21947816

[B16] Li Y. Reid M. B. (2001). Effect of tumor necrosis factor-[alpha] on skeletal muscle metabolism. Curr. Opin. Rheumatol. 13, 483–487. 10.1097/00002281-200111000-00005 11698724

[B17] Mackey A. L. Kjaer M. Dandanell S. Mikkelsen K. H. Holm L. Døssing S.. (2007). The influence of anti-inflammatory medication on exercise-induced myogenic precursor cell responses in humans. J. Appl. Physiol. 103, 425–431. 10.1152/japplphysiol.00157.2007 17463304

[B18] Miller B. F. Gruben K. G. Morgan B. J. (2000). Circulatory responses to voluntary and electrically induced muscle contractions in humans. Phys. Ther. 80, 53–60. 10623959

[B19] Peake J. Nosaka K. K. Muthalib M. Suzuki K. (2006). Systemic inflammatory responses to maximal versus submaximal lengthening contractions of the elbow flexors. Exerc. Immunol. Rev. 12, 72–85. 17201073

[B20] Peake J. Nosaka K. K. Suzuki K. (2005). Characterization of inflammatory responses to eccentric exercise in humans. Exerc. Immunol. Rev. 11, 64–85. 16385845

[B21] Peake J. Peiffer J. J. Abbiss C. R. Nosaka K. Okutsu M. Laursen P. B.. (2008). Body temperature and its effect on leukocyte mobilization, cytokines and markers of neutrophil activation during and after exercise. Eur. J. Appl. Physiol. 102, 391–401. 10.1007/s00421-007-0598-1 17962974

[B22] Peterson J. M. Feeback K. D. Baas J. H. Pizza F. X. (2006). Tumor necrosis factor-α promotes the accumulation of neutrophils and macrophages in skeletal muscle. J. Appl. Physiol. 101, 1394–1399. 10.1152/japplphysiol.01453.2005 16840574

[B23] Piers A. Lavin T. Radley-Crabb H. Bakker A. Grounds M. Pinniger G. (2011). Blockade of TNF-α *in vivo* using cV1q antibody reduces contractile dysfunction of skeletal muscle in response to eccentric exercise in dystrophic mdx and normal mice. Neuromuscul. Disord. 21, 132–141. 10.1016/j.nmd.2010.09.013 21055937

[B24] Rice T. Chantler I. Loram L. C. (2008). Neutralisation of muscle tumour necrosis factor alpha does not attenuate exercise-induced muscle pain but does improve muscle strength in healthy male volunteers. Br. J. Sports Med. 42, 758–762. 10.1136/bjsm.2007.038067 17717057

[B25] Smith L. Anwar A. Fragen M. Rananto C. Johnson R. Holbert D. (2000). Cytokines and cell adhesion molecules associated with high-intensity eccentric exercise. Eur. J. Appl. Physiol. 82, 61–67. 10.1007/s004210050652 10879444

[B26] Tallone T. Turconi G. Soldati G. Pedrazzini G. Moccetti T. Vassalli G. (2011). Heterogeneity of human monocytes: an optimized four-color flow cytometry protocol for analysis of monocyte subsets. J. Cardiovasc. Transl. Res. 4, 211–219. 10.1007/s12265-011-9256-4 21308491

[B27] Townsend J. R. Fragala M. S. Jajtner A. R. Gonzalez A. M. Wells A. J. Mangine G. T.. (2013). β-Hydroxy-beta-methylbutyrate (HMB)-free acid attenuates circulating TNF-alpha and TNFR1 expression postresistance exercise. J. Appl. Physiol. 115, 1173–1182. 10.1152/japplphysiol.00738.2013 23908318

[B28] Tseng C. Y. Lee J. P. Tsai Y. S. Lee S. D. Kao C. L. Lai C.. (2013). Topical cooling (icing) delays recovery from eccentric exercise-induced muscle damage. J. Strength Cond. Res. 27, 1354–1361. 10.1519/JSC.0b013e318267a22c 22820210

[B29] Urso M. L. (2013). Anti-inflammatory interventions and skeletal muscle injury: benefit or detriment? J. Appl. Physiol. 115, 920–928. 10.1152/japplphysiol.00036.2013 23539314

[B30] Vanderthommen M. Soltani K. Maquet D. Crielaard J. Croisier J. (2007). Does neuromuscular electrical stimulation influence muscle recovery after maximal isokinetic exercise? Isokinet. Exerc. Sci. 15, 143–149.

[B31] Zaldivar F. Wang-Rodriguez J. Nemet D. Schwindt C. Galassetti P. Mills P. J.. (2006). Constitutive pro-and anti-inflammatory cytokine and growth factor response to exercise in leukocytes. J. Appl. Physiol. 100, 1124–1133. 10.1152/japplphysiol.00562.2005 16357073

